# 

**Published:** 1781-04

**Authors:** 


### SECTION IV.

### Monthly Catalogue.

I. *A Treatise on Sympathy, in two Parts. By* Seguin Henry Jackson, M. D. &c. 8vo. London. 1781. 274 *pages.*

THIS work is written in the form of aphorisms.—To the three general heads or divisions of physic (physiology, pathology, and therapeiea) usually enumerated by systematic writers, our author has added a fourth, but without venturing to give it a name. His words are as follows: " I shall take the liberty of " adding a fourth, very lately discovered, but " of sufficient importance to claim a place and " engage our attention; namely, the doctrine " of restoring animation, or the vital principle, " when apparently lost, or, * * * * * *."— We leave it to the ingenious adepts in the art of guessing to determine what our author means by his six asterisks.

Speaking of the subject of his work, sympathy, he observes, (p. 5.) that " she even gave us " life; she breathed into us when born into the

" world;

" world; ſhe preſerves our lives while in it, " guards us againſt the diſeaſes of it, proves " fatal to us when in exceſs, and when life has " not been too long apparently extinct, is capa- " ble of reſtoring us to the world again." And in another place (p. 14) he defines her to be " nature's hand-maid in the conſtitution and " government of the animal œconomy." In p. 28, we are told, that " an animal may have " an agreeable feeling, yet no ſenſation of it." In p. 30, he informs us, that " a man in perfect " health and vigour, who lives well, &c. ſhall " be ſo captivated by the beauty of one woman, " or the good ſenſe and underſtanding of ano- " ther, as to excite a *ſympathy of impreſſion* from " the ſight of the one, and *ſympathy of conſciouſ-* " *neſs* from the company of the other; but that " on the contrary, if he be lean, ſtarving, &c. " no ſuch impreſſion or affection ſhall be pro- " duced." Theſe ſympathies of impreſſion and conſciouſneſs from external objects require, as we are told in the ſame page, " a certain degree " of warmth, a certain ſupply of nutriment to " produce them."

Treating of univerſal ſympathy our author remarks, (p. 50) that it is " greater, if the in- " jury is done to a part far remote from the

" ſup-

" ſupport of life, the heart, than if the part in-

" jured be nearer, provided the injury be the

" ſame. The animal machine is then more

" conſcious of inability and more alarmed.

" Ex. gr. If an injury be done to the toes, the

" conſtitution becomes more affected and diſ-

" turbed than if a ſimilar injury had been done

" to the ſhoulder."

In p. 74, the author ſpeaks of a kind of ſtone brought out of the " Weſt-Indies, which is ſaid " to have a peculiar property of diſcharging " gravel, and of diſſolving the ſtone; inſomuch " that when laid to the wriſt, it has ſo forcibly " expelled urine and gravel, by its violent man- " ner of operating, that the ſick perſon has been " glad to remove it."

In noticing the ſympathy between the ſoles of the feet and the head, he takes occaſion to obſerve, that " formerly a phyſician, who wiſhed " to appear myſtical, preſcribed for the cure of " the rheum, that the patient ſhould walk con- " tinually upon a camomile alley, (ſuch was " the old language) meaning thereby that he " ſhould put camomile into his ſocks." And in the page following, in order to illuſtrate the ſympathy which the author ſuppoſes to prevail between the hands and the heart, we are told, that "eggs of alabafter, and balls of chryftal, "have been held in the hand, in order to ap- "peafe the fury of a febrile heat."

The fourth fection of chapter IV, is entitled, "*Sympathy, when the province of the phyfician and* "*when of the furgeon.*" As it confifts of only two fhort aphorifms, we fhall tranfcribe the whole of it. "Difeafes with their fympathies "are either local or univerfal. If fympathy be "either particular or univerfal, and the caufe be "known, it falls under the care of the *medical* "furgeon; but if the caufe be not known, or if "it be not even known that a caufe exifts, then "the fympathy becomes the province of a phy- "fician, more efpecially if it is univerfal. . . . . . "Local difeafes in all cafes come more properly "under the care of the furgeon; when univer- "fal, they fall under the province of the phy- "fician."

Warm applications to the furface, according to our author, act, not by relaxing the integu- ments, or by increafing determination to the fkin, as many have fuppofed, but by fympathy. "A man (fays he) meets with a diflocation, he "is ordered to be put into warm water to be "relaxed; but the ligaments will not become "a bit moifter, though you was to foak him to

"eter-

" eternity." He laments, and feelingly too, that an animal body labouring under the want of any free motion, is lefs happy than the hinge of a door. " Unfortunately (fays he) for the " animal body, oil cannot penetrate beyond the " furface, to effect mechanically the inward " parts; fortunately for the hinge, oil may pe- " netrate into all its moving apparatus."

In defcribing the effects of the paffions (p.96) the author obferves, that, firft, " Fear caufeth " palenefs, trembling, the ftanding of the hair " upright, ftarting, and fcreeching. 2. Grief " and pain *caufeth* fighing, fobbing, groaning, " fcreaming, and roaring: they alfo caufe tears, " diftorting of the face, grinding of the teeth, " and fweating. 3. Joy caufeth a chearfulnefs " and vigour in the eyes; finging, leaping, " dancing, and fometimes tears. 4. Anger pro- " duces palenefs in fome, and the going and " coming of the colour in others: alfo trem- " bling in fome, fwelling, foaming at the mouth, " ftamping, and bending of the fift. 5. Slight " difpleafure, or diflike, caufes fhaking of the " head, and knitting of the brows. 6. Shame " caufeth blufhing and cafting down of the eyes. " 7. Pity caufes fometimes tears and a caft of " the eyes afide. 8. Wonder caufeth aftonifh-

" ment,

" ments, and an immoveable posture of the

" body, casting up of the eyes to heaven, and

" lifting up of the hands. 9. Laughing, though

" hardly to be considered as a passion, since it

" is produced by an affection of the mind,

" causeth a dilatation of the mouth and lips;

" a continued expulsion of the breath; with a

" loud noise, which maketh the interjection of

" laughing, shaking of the breast and sides,

" and running of the eyes with water, if it be

" violent and continued. 10. Lust causes a

" flagrancy in the eyes and priapism."

In p. 99, we are informed, that " the passions

" of the mind are occasionally infective. Thus,

" (adds our author) fear and shame are some-

" times very suddenly so. We frequently may

" have occasion to see that the starting of one

" will make another ready to start. Again,

" when one man is out of countenance in com-

" pany, others will often blush in his behalf."

The eyes, he observes, are naturally disposed to move in concert, so that " when one eye

" moveth towards the nose, the other eye mov-

" eth from the nose." But it seems that custom will destroy this natural sympathy, so that (as our author expresses himself) " some people

" will squint when they will."—This leads him

 to to offer a caution to mothers and nurſes, "not "to ſuffer infants to ſit with a candle placed "behind them, for both their eyes will diſpoſe "to move outwards, as affecting to ſee the "light of the candle, which may bring on the "habit of ſquinting."

Speaking of the ſtomach (p. 145) he obſerves, that "ſome have compared it to a mill, others "to a ſtewing-pot, others to a wort trough, "when all the while it muſt have appeared "that it was neither a mill, nor a ſtewing-pot, "nor a wort trough, nor any thing elſe but a "ſtomach." In page 147, it is ſpoken of as "*the ſeat of ſympathy*, *the throne of ſenſibility*, to "which all the other functions of the ſyſtem "look up."

From theſe few paſſages ſelected from different parts of the work, the reader will be enabled to judge of the author's abilities as a medical writer.

2. Cours complet de Chymie economique et pratique ſur la manipulation et la fermentation des vins, diviſé par leçons, avec le decret de la Faculté de medecine de Paris, &c. par *M. Maupin*, auteur de l'art des vins, &c. *i. e.* A Complete Courſe of œconomical and practical chymiſtry, on the manipulation and fermentation of wines, divided into leſſons, with the decree of the the College of Phyficians at Paris, by *Monfieur Maupin*, author of the Art of making Wines, &c. 8vo. Paris, 1780. 42 pages.

3. Memoire fur l'Electricité Medicale, et hiftoire du traitement de vingt malades traités et la plupart gueris par l'Electricité. Par *M. Mafars de Cazelles*, Docteur en Medecine de l'Univerfité de Montpelier, agregé à la faculté de Touloufe, de l'Academie de Beziers, correfpondant de la Societé Royale de Medecine de Paris, Medecin à Touloufe. *i. e.* A Memoir on Medical Electricity, with an account of twenty patients treated, and for the moft part cured by Electricity. By *M. Mafars de Cazelles*, M. D. &c. 8vo. Paris, 1780. 122 pages.

4. Dictionnaire de Phyfique. Par *M. Sigaud de Lafond*, Profeffeur de Phyfique experimentale, Membre de la Societé Royale des Sciences de Montpellier, des Academies de Baviere, de Valladolid, de Florence, de Peterfbourg, &c. *i. e.* A Dictionary of Natural Philofophy. By *M. Sigaud de Lafond*, Profeffor of Experimental Philofophy, &c. Paris, 1780. 4 volumes octavo, of about 700 pages each, with copper-plates.

The reputation of M. Lafond is already well eftablifhed as a philofophical writer, by his *Cours de Phyfique*, in 4 volumes octavo; his tranflation of Muſſenbroeck in three volumes, quarto, and his other works. The preſent performance appears to be extremely well executed, and of courſe cannot but prove a very uſeful work.

5. Eſſai ſur la generation de l'Homme, par *M. Calmé*, Docteur en Mèdecine à Sezanne en Brie. *i. e.* An Eſſay on the generation of man, by *M. Calmé*, M. D. at Sezanne in Brie. 8vo. Paris, 1781, 47 pages.

Our author thinks with Ariſtotle, that the male furniſhes the germen, and that the female only receives and nouriſhes it. His reaſoning is very often obſcure, and being founded wholly on hypotheſes, and thoſe not very plauſible ones, does not ſeem to be deſerving of much attention.

6. Avis au peuple ſur les hernies ou deſcentes, par *M. Foujols*, Docteur en Med. de l'univerſité de Montpellier, medecin ordinaire du grand conſeil, ci devant med. et chir. major de la premiere compagnie des mouſquetaires, *i. e.* Advice to the people concerning hernias or ruptures, by *M. Foujols*, M. D, &c. 12mo. Paris, 1781. 168 pages.

The author has treated his ſubject in a very ſuperficial manner. He diſtinguiſhes different ſpecies of ſtrangulation, ſuch as the inflammatory, tory, the ſpaſmodic, &c. each of which he obſerves requires a different treatment; but he has omitted to point out the ſymptoms that characterize each of theſe ſpecies.

7. Diſſertatio medica inauguralis de obeſitate nimiâ et morbis inde oriundis. Auctore *Fred. Chriſt. Gul. Ebart.* 4to. Gottingen, 1780.

A female child of ſingular bulk, whom our author had occaſion to ſee, ſeems to have been his chief inducement to write on this ſubject. This girl, at the age of three years, weighed ſeventy-five pounds, was near three feet high, and meaſured four feet round her body. Her breaſts were equal in ſize to thoſe of a girl of eighteen years. She was unable to retain her urine, and the tunica conjunctiva of her eyes was of a yellowiſh tinge.

8. Opuſcules chymiques & phyſiques de *M. Bergman*, profeſſeur de chymie à Upſal, recueillis, revus, et augmentés par lui-meme; traduits par *M. de Morveau*, avec des notes. *i. e.* Eſſays chemical and philoſophical, by *T. Bergman*, profeſſor of chemiſtry at Upſal, collected, reviſed, and augmented by himſelf; tranſlated by *M. de Morveau*, with notes. Vol. Iſt. 8vo. Dijon, 1780.

This

This is an accurate tranflation, and the notes are extremely judicious. M. de Morveau promifes foon to favour the public with a fecond volume.

9. Praktifche Materia Medica, von *C. F. Mellins*. 8vo. Altenburg, 1779, 303 pages.

This work contains fhort accounts of fuch fimple medicines as have been found really efficacious in practice.

10. *J. G. Zinn*, M. D. Defcriptio anatomica oculi humani iconibus illuftrata, nunc altera vice edita, et neceffario fupplemento novifque tabulis aucta ab *H. A. Wrifberg*, M. D. et anatom. prof. Goettingæ, 4to. 1780, 248 pages, with 7 copper-plates.

11. *J. H. Rahn*, M. D. Adverfaria medico practica, Tomus I. 8vo. Turici, 1779, 408 pages.

This firft volume contains only one article, which is the *cinchona officin.* L. or Peruvian Bark. On this fubject the author with great induftry and judgment has collected every thing that has been written by former writers concerning it. He firft gives its natural hiftory, and then treats of its medicinal virtues.

12. Effai fur les eaux minerales ferrugineufes de Spa, *i. e.* An effay on the chalybeate mineral waters waters of Spa, by *M. Sanberg*, M. D. phyſician at Spa. 12mo. Liege, 1780, 210 pages.

13. Doctoris *Franciſci Cremadells* in Archynoſocomio ſancti ſpiritus urbis medici ſecundarii Nova Phyſiologiæ Elementa. 8vo. Romæ, 164 pages.

This work is founded chiefly on the doctrines delivered by a M. Barthez at Montpellier, who aſcribes all the phænomena of animal life to a *principium vitale*, as Van Helmont did to his *Archeus*. Thus, in the volume before us we are told, that the cauſe of reſpiration is owing to this principle, which produces in the new-born animal what our author calls "automatica "aeris appetentia," a ſort of inſtinctive craving for air, which is perpetually renewed during life. He ſeems to think, however, that by degrees the *principium vitale* may be reconciled to the carrying on life without reſpiration, and accordingly informs us page 79, that "Exempla non "deſunt quibus probabile redditur ferè omninò "tolli poſſe neceſſitatem aeris reſpiratione reno-"vandi quin vita adimatur."

14. Obſervations medical and political, on the ſmall-pox and inoculation, and on the decreaſe of mankind at every age; with a comparative view of the diſeaſes moſt fatal to London during during ninety years. Including an attempt to demonſtrate in what manner London may ſave near two thouſand, Great Britain and Ireland between twenty and thirty thouſand, and Europe about three hundred and ninety thouſand lives annually. By *W. Black*, M. D. 8vo. London, 1781, 202 pages.

This pamphlet appears to be the production of ſome friend to the Diſpenſary lately eſtabliſhed for Inoculating the poor at their own habitations, the chief aim of the writer ſeeming to be to oppoſe Baron Dimſdale's objections to this plan; but we do not perceive that he has advanced any new arguments on the ſubject. His obſervations on the decreaſe of mankind, and on the Bills of Mortality, are taken from Dr. Price and other political writers. His ſcheme for ſaving three hundred and ninety thouſand lives annually to Europe, is ſimply a propoſal to eradicate the ſmall-pox from Europe by a general inoculation; "to baniſh it to its original birth-"place in either Arabia or India, and to ſet up "barriers againſt its return and communication, "as in caſes of plague."

